# Examining Public Awareness of Ageist Terms on Twitter: Content Analysis

**DOI:** 10.2196/41448

**Published:** 2023-09-11

**Authors:** Emily Schramm, Christopher C Yang, Chia-Hsuan Chang, Kristine Mulhorn, Shushi Yoshinaga, Jina Huh-Yoo

**Affiliations:** 1 College of Medicine, Drexel University, Philadelphia, PA, United States; 2 Department of Information Science, College of Computing and Informatics, Drexel University, Philadelphia, PA, United States; 3 Health Administration Department, Drexel University, Philadelphia, PA, United States; 4 Westphal College of Media Arts and Design, Drexel University, Philadelphia, PA, United States

**Keywords:** social media, informatics, aging, ageism, public, COVID-19, disease, language, older adults, Twitter, elderly, term, terminology, pandemic, tweets

## Abstract

**Background:**

The World Health Organization, the Centers for Disease Control and Prevention, and the Gerontological Society of America have made efforts to raise awareness on ageist language and propose appropriate terms to denote the older adult population. The COVID-19 pandemic and older adults’ vulnerability to the disease have perpetuated hostile ageist discourse on social media. This is an opportune time to understand the prevalence and use of ageist language and discuss the ways forward.

**Objective:**

This study aimed to understand the prevalence and situated use of ageist terms on Twitter.

**Methods:**

We collected 60.32 million tweets between March and July 2020 containing terms related to COVID-19. We then conducted a mixed methods study comprising a content analysis and a descriptive quantitative analysis.

**Results:**

A total of 58,930 tweets contained the ageist terms “old people” or “elderly.” The more appropriate term “older adult” was found in 11,328 tweets. Twitter users used ageist terms (eg, “old people” and “elderly”) to criticize ageist messages (17/60, 28%), showing a lack of understanding of appropriate terms to describe older adults. Highly hostile ageist content against older adults came from tweets that contained the derogatory terms “old people” (22/30, 73%) or “elderly” (13/30, 43%).

**Conclusions:**

The public discourse observed on Twitter shows a continued lack of understanding of appropriate terms to use when referring to older adults. Effort is needed to eliminate the perpetuation of ageist messages that challenge healthy aging. Our study highlights the need to inform the public about appropriate language use and ageism.

## Introduction

Ageism has been defined as the “ideas, attitudes, beliefs, and practices on the part of individuals that are biased against persons or groups based on their age” [1] and “stereotyping and discrimination against individuals or groups based on their age” [2]. Ageism stems from everyday language that portrays older adults as a burden on society. Older adults are associated with the derogatory terms “geriatric,” “geezer,” and “senior citizen” while being inappropriately described as “adorable,” “dried up,” or “grumpy” [3]. This language perpetuates a stigma surrounding older adults in our society, such as the perspective that older adults are frail and incompetent [4] or that they are out of touch and burdensome [2]. Many perceive aging as a problem that needs to be fixed, leading to the segregation and social exclusion of older adults.

Ageism influences the physical and mental health of older adults [5]. Research has shown that internalized ageism is associated with a lower life expectancy, high blood pressure, reduced self-esteem, diminished risk taking, and decreased motivation [6]. Summers [1] further stated that the functional health of older individuals can worsen over time as a result of insults and negative images [1]. This is particularly detrimental considering the positive impact this population has on our society. For example, older individuals contribute significantly to the economy as they have additional free time and a more flexible income [7]. Furthermore, older adults stay active in their communities through volunteering, activism, advocacy, and nurturing [8].

The isolation of older adults from the rest of society has become more evident during the COVID-19 pandemic. Only 5% of Chinese people aged 60 years and older use the internet on a regular basis [9]. During a pandemic, older adults who do not know how to use technology to shop for groceries, socialize, order medications, or educate themselves about the virus may face difficulties [9]. Moreover, as governments try to establish a new “normal,” older adults are being increasingly isolated. During the earlier part of the pandemic, the Chinese Center for Disease Control and Prevention cautioned older adults against traveling while the rest of the public was urged to “go out and resume spending” [9]. There is a false sense of security for younger members of our society because “COVID-19 is life-threatening, but mostly for the older adults and those with underlying conditions” [10]. This perspective propagates ageism, which impacts everyone in due time as no one can avoid aging.

Due to the negative impact caused by the public’s use of ageist language, the editor of the *Journal of Gerontological Social Work* announced the replacement of “the elderly” with “older adults” when “vulnerable” is used [11]. The Gerontological Society of America has promoted the Reframing Aging Project’s recommendations to use “older people” or “older adults” instead of “senior” and “the elderly” as well as inclusive “we” and “us” terms [12]. Current efforts to combat ageism from the World Health Organization and the Gerontological Society of America are examples of widespread campaigns to address this critical problem, with a target audience comprising largely researchers and those working in aging services. The American Association of Retired Persons (AARP) also created a public-facing effort to guide the public toward more inclusive language that does not segment older adults. Terms such as “older persons,” “older people,” “older adults,” “older patients,” “older individuals,” “persons 65 years and older,” and “the older population” are preferred. Terms such as “seniors,” “elderly,” “the aged,” “aging dependents,” and similar “othering” terms are not recommended because they connote a stereotype and suggest that older adults are not part of society but are a group apart [13,14]. The AARP has been promoting a campaign to “disrupt ageism” for at least 3 years now, with recommendations for employers, employees, and others to use language that is respectful and does not reinforce stereotypes and myths about older persons.

Studies on social media have successfully exposed societal norms and biases and have shown how social media may be influencing societal norms, social movements, and individuals’ perceptions. Its large-scale ability to propagate ideologies has broad implications for creating and spreading harmful perceptions about racism, sexism, and ageism, often coupled with political influence [15]. Accordingly, researchers have used Twitter data to assess the propagation of ideologies related to health, wellness, politics, and public health at the societal and individual levels [16-19].

The impact of social media in a number of areas has been documented at the individual and societal levels: political influence [15,17,20,21], individuals’ mental health affected by sexist and racist discourse online [16,22], and public health messages [19,23,24]. The prevalence of ageist content and the use of unsuitable language are troubling, not just for older adults but for everyone, given that we all age. The percentage of older adults (≥65 years) using social media has grown from 11% to 45% during the 2010-2021 period [25], and the negative public discourse will be harmful to those exposed to ageist content. Existing studies inform our understanding of public discourse and the ways through which we can move forward to resolve any conflicts and tensions.

Building on previous works, we investigated how the public used ageist language on Twitter, particularly during the COVID-19 pandemic, given the increases in ageist content observed during this time period [26]. We aimed to answer the following 2 research questions (RQs):

- RQ1: What is the prevalence of improper terms (eg, “old people” and “elderly”) referring to older adults on Twitter?
- RQ2: How are the terms referring to older adults associated with ageist and antiageist content?

To answer these questions, we collected 60.32 million tweets with hashtags related to COVID-19. We then conducted a mixed methods study comprising a content analysis and a descriptive quantitative analysis.

## Methods

### Twitter Data Collection and Keyword Search

We collected Twitter posts between March and July 2020 that included these COVID-19–related hashtags: #COVID, #Sars-Cov, and #COVID19. We then subsampled posts that used ageist language as well as language that may indicate discussion on ageist discourse. To subsample Twitter posts that used ageist language, we identified a list of ageist language keywords aggregated from existing articles. In addition, research team members who are experts in ageism also brainstormed for keywords. We then searched these keywords on the Twitter website. Using the first 20 posts we retrieved, we assessed whether the posts referred to older adults. We then narrowed down the list of keywords, discarding those that resulted in irrelevant posts. The final keywords were “old people,” “elderly,” “older adult,” “ageist,” and “ageism.” Other keywords, such as “senior,” for instance, retrieved too many irrelevant posts, such as those related to “high school seniors.” Thus, we excluded tweets that used such terms from the analysis.

### Qualitative Analysis

With the collected data, we used two methods to address the two RQs. First, we developed a codebook for a qualitative content analysis based on an existing survey instrument that measures ageist perception or ageist content. Several instruments exist that measure ageism: the Ambivalent Ageism Scale [27], the Ageism Survey [28], the Fraboni Scale of Ageism (FSA) [29], and the Competence and Warmth Scale [30]. Among these scales, we used the FSA, given its high citation, comprehensiveness, and inclusivity of ageist concepts when compared to the other surveys. We thematized the FSA survey questions into three categories: (1) the perception that older individuals cannot make good decisions, (2) the perception that older individuals are a burden on society, and (3) the devaluing of older individuals’ lives. We then applied these themes as codes for ageist content. We also included a theme to code antiageist content that criticizes ageist messages.

We randomly selected 150 tweets from the Twitter data. We randomly divided these tweets into 3 sets of 50 tweets and distributed the data to 3 coders. Using the codebook, 3 individuals coded 50 randomly selected tweets and 1 coder coded all 150 tweets. Given the implicit nature of the Twitter data (eg, posts can indicate sarcasm), it was challenging to establish high interrater reliability. Instead, we coded a common set of 20 tweets as a group, and using the agreed-upon codebook, we individually coded the assigned tweets. We then negotiated any disagreements.

### Quantitative Analysis

For the descriptive quantitative analysis, we first counted the total posts that contained the keywords mentioned above. We also counted frequencies of ageist versus antiageist posts from the qualitative findings to understand the relationship between ageist content and terminology used to denote older adults. Specifically, we wanted to understand whether posts that included ageist terms were intended to be ageist and whether posts that included appropriate terms were nonageist.

### Ethical Considerations

All data reviewed are publicly available and were collected through the Twitter API (application programming interface) and deidentified. Therefore, no ethical review or approval was deemed necessary.

## Results

### Use of Ageist Terms (RQ1)

As shown in Table 1, out of 60.32 million tweets, the term “elderly” occurred in 32,700 tweets (0.05%), “old people” occurred in 26,230 tweets (0.04%), and “older adult” occurred in 11,328 tweets (0.02%).

**Table 1.** The prevalence of terms referring to older adults on Twitter in tweets related to COVID-19 (N=60,320,000).

Keyword	Frequency^a^, n (%)	Examples
“elderly”	32,700 (0.05)	“Keep your elderly folks from flying. If the CDC advised the elderly population to try to stay indoors as much as possible the elderly should not be exposing themselves to airports filled with people or get on a plane where the air is recycled. This goes for (1 #CoronoaVirus)” “It is but if ur elderly or got elderly family or existing diseases affected by flu or weak immune system then its bad”
”old people”	26,230 (0.04)	“false alarm everybody turns out the coronavirus only kills old people” “Young people on the ship MORE likely to have symptoms. We were fed another lie. More old people got infected because old ppl like cruises. #COVID19 #SARSCoV2 #coronavirus #outbreak #pandemic #USA #COVID19 #CDC #NYC #coronavirususa #Covid_19 #COVID2019 #COVID2019 #coronavirusus” “Old people disproportionately die from #COVID19”
”older adult”	11,328 (0.02)	“New CDC guidance says older adults should ‘stay at home as much as possible’ due to coronavirus” “Advice from @WHO about keeping safe in older adults or at risk groups People, chat to your relatives now. Look after them. Wash your hands” “Should older adults stay home to avoid coronavirus? Heres what health experts say - Macon Telegraph #news #feedly”

a Number of tweets that included the keyword.

### Terms Referring to Older Adults and Ageist and Antiageist Content (RQ2)

Next, we investigated whether each term referring to older adults in tweets promoted or spoke against ageism. Table 2 describes the frequency results.

**Table 2.** The qualitative coding results identifying ageist content.

Keywords		Code			Tweets, n		
		C1^a^	C2^b^	C3^c^	Ageist tweets	Tweets against ageism	Neutral tweets
**Keyword against ageism, n (%)**							
	“ageism” (n=30)	0 (0)	0 (0)	0 (0)	0 (0)	23 (77)	7 (23)
	“ageist” (n=30)	1 (3)	2 (7)	4 (13)	7 (23)	21 (70)	2 (7)
**Proper term, n (%)**							
	“older adult” (n=30)	3 (10)	1 (3)	1 (3)	5 (17)	7 (23)	18 (60)
**Improper term, n (%)**							
	“elderly” (n=30)	1 (3)	5 (17)	7 (23)	13 (43)	12 (40)	3 (10)
	“old people” (n=30)	4 (13)	2 (7)	6 (20)	22 (73)	5 (17)	3 (10)

a C1: Does this tweet suggest older individuals cannot make good decisions?

b C2: Does this tweet suggest older individuals are a burden on society?

c C3: Does this tweet devalue older individuals’ lives?

#### “Old People” Tweets

The majority of the tweets that contained the term “old people” were flagged as ageist (22/30, 73%), specifically in terms of devaluing older individuals’ lives (16/30, 53%). These tweets included those linking the vulnerability of older adults to COVID-19 as a positive outcome of the pandemic. For instance, a tweet with “old people” as a keyword said, “how old is the editor/journalist. I heard the novel coronavirus love old people so hard. What I am hoping on top of my head is all old and old minded people be wiped out by this old-loving virus.” Four tweets with the term “old people” suggested older individuals cannot make good decisions. These tweets criticized “old people” as being unable to make good decisions in terms of voting. Two tweets included content that suggested older adults are a burden on society.

#### “Elderly” Tweets

Of the tweets that included the term “elderly,” 43% (13/30) were ageist whereas 40% (12/30) were against ageism. The majority of the ageist tweets with the term “elderly” included content that suggested older individuals are a burden on society (5/30, 16.7%) and devalued older individuals’ lives (7/30, 23.3%). For example, extremely hostile tweets containing the term “elderly” were shared, including the following: “The elderly are a drag on the world economy. Covid19 preferentially kills the elderly. Illuminati developed Covid19 to prune the elderly population.” On the other hand, antiageist tweets with the term “elderly” contained content that questioned how the threshold of “elderly” was defined and shared criticisms against ageist incidences around the world. An example tweet said: “ok so all these fucking attacks on elderly nfluen in San Francisco etc are disgusting. Ya’ll need to sit your ass down, get re-educated and respect THE FUCKING ELDERLY. Not just elderly, but seriously. What gives you the RIGHT to assault, rob or humiliate a person. #COVID19.”

#### “Ageism” and “Ageist” Tweets

The majority of the discourse that involved the terms “ageism” and “ageist” was about raising awareness of ageism. These tweets critiqued the widely circulating perspectives of COVID-19–related ageist tweets on the vulnerability of older adults and individuals with chronic illnesses and the reduced danger of COVID-19 given that it “only kills old people.” Multimedia Appendix 1 provides frequencies and examples of Twitter content that included “ageism” and “ageist” terms in our data set.

#### “Older Adult” Tweets

While the term “older adult” is an aging-friendly term, we still observed a few tweets (5/30, 17%) associated with ageist content. For instance, themes seen in other ageist tweets were observed in “older adult” tweets (eg, older adults cannot make good political decisions: “You heard it here, folks. Let the young people handle voting. No need for older adults to risk it at their local polling location!”). The majority of the tweets were neutral, meaning that the tweet was neither ageist nor specifically arguing against being ageist. “Older adult” tweets were often retweets of news articles on older adults related to the COVID-19 pandemic (eg, “Older adults should ‘stay at home as much as possible’ due to coronavirus, CDC says”).

## Discussion

### Principal Findings

We found that the use of ageist terms was prevalent on Twitter. Twitter users also used ageist terms to criticize ageist messages, showing a lack of understanding of the appropriate use of terms when referring to older adults. Highly hostile ageist content against older adults came from tweets that denoted older adults with derogatory terms.

### Comparison to Prior Work

Ageism has been heightened during the COVID-19 pandemic, and researchers have highlighted the hostile messages being propagated through social media against older adults and their role in society [31-34]. Accordingly, this was an opportune time for researchers to assess what language is being used and how this language is associated with ageist messages.

The terms “old people” and “elderly” were more prevalent than “older adult” in tweets. This finding shows a continued use of ageist terms despite the efforts of various organizations to raise awareness about not “othering” older adults. In addition, tweets with the term “elderly” showed a high percentage of antiageist content, which also suggests a lack of awareness of recommended terminology and of the derogatory nature of the term “elderly” as deemed by the AARP.

### Strengths and Limitations

Our paper uniquely contributes to the field of aging using social media data and qualitative and quantitative methodologies to assess the public’s use of common ageist terms. This study has a few limitations. First, the terms we used to assess ageist term use were not exhaustive. Such limitation came from the fact that the excluded terms were used in multiple ways depending on the context (eg, “senior” referring to senior housing rather than to older adults). Thus, we limited our use of the terms for analysis to those that were explicitly referring to older adults. Although using exhaustive terms would have generated more complete results, we were able to address our RQs with the terms we identified. Second, Twitter content can often convey sarcasm, which can result in interpretive errors. We engaged all coauthors of this manuscript to discuss and converge on the final interpretation. Lastly, the qualitative analysis was limited to a subset of the Twitter data set due to logistical feasibility (eg, time and resources needed for manual coding). However, this is a common practice in follow-up qualitative research to give richer nuance and context to quantitative results.

### Future Directions

We suggest several future directions for this study. First, we can develop a training data set based on our qualitative research results to automate the identification of ageist terms on Twitter and perform a larger-scale study on the RQs. Second, a repeated follow-up study with post–COVID-19 Twitter data will help us understand how term usage has evolved over time. Third, using social network analysis, we can identify how influential Twitter users use ageist terms and how impactful their tweets are among their followers over nonageist content. Lastly, future work should examine how often ageist content is challenged and critiqued by antiageist responses. Public policies, organizations, and the technology industry should develop creative solutions to detect harmful content and educate the public on appropriate terminology use and how to change harmful perceptions of older adults.

### Conclusions

The COVID-19 pandemic and older adults’ vulnerability to the disease have perpetuated hostile ageist discourse on social media. This is an opportune time to understand the prevalence and use of ageist language and discuss the ways forward. From examining tweets related to COVID-19, we were able to uncover the prevalence of the ageist terms used and the contexts in which these terms were used. The findings showed a continued lack of understanding among the public on the appropriate use of terms that refer to older adults. This paper emphasizes the need to put more effort into eradicating the perpetuation of ageist messages that challenge healthy aging.

## Supplementary material

10.2196/41448 Multimedia Appendix 1 Tweets that included the terms “ageism” and “ageist.”

## Abbreviations

AARP: American Association of Retired Persons
API: application programming interface
FSA: Fraboni Scale of Ageism
RQ: research question
