# Supplementary material for: Examining Public Awareness of Ageist Terms on Twitter: Content Analysis
Source: JMIR Aging. 2023 Sep 11;6:e41448. doi: 10.2196/41448 (PMC10507520; doi:10.2196/41448)
Supplement: Multimedia Appendix 1 [file aging-v6-e41448-s001.docx]

# Appendix 1. The discourse that included the term “ageism” occurred in N=2,778 (0.005%) tweets, and the term “ageist” occurred in N=672 tweets (0.001%).

| Appendix 1. The table shows the prevalence of terms referring to ageism on Twitter related to COVID (N=60.32M). | | |
| --- | --- | --- |
| Keywords | Frequency^a^ n, (%) | Examples |
| Ageism | 2,778 (0.005) | - “What the coronavirus crisis has taught me is that ageism is real. It mostly kills old people.” - “If you are feeling flippant about #COVID19, please think about how that attitude was potentially precipitated by ableism or ageism and do better.” |
| Ageist | 672 (0.001) | - “COVID19 is ageist.” - “If youre tweeting about #COVID19 #coronavirus, please mind your words. A large percentage of the country and world lives with chronic health challenges or is over 50. The dont freak out, youll be fine unless... is invalidating, ableist and ageist.” |
| ^a^frequency: number of tweets including the keyword | | |
